# The larvae of the European species of genus *Apataniana* Mosely, 1936 (Trichoptera, Apataniidae): descriptions, key and ecology

**DOI:** 10.3897/zookeys.586.7758

**Published:** 2016-05-04

**Authors:** Johann Waringer, Hans Malicky

**Affiliations:** 1 Department of Limnology and Bio-Oceanography, Faculty of Life Sciences, University of Vienna, Austria; 2 Sonnengasse 13, A-3293 Lunz am See, Austria

**Keywords:** Description, distribution, larvae, identification, ecology

## Abstract

This paper describes the previously unknown or insufficiently known larvae of *Apataniana hellenica*, *Apataniana stropones* and *Apataniana vardusia*. Species association was enabled by the fact that the three micro-endemic *Apataniana* larvae are restricted to Greece and the only Apataniidae species recorded in European ecoregion 6 (Hellenic Western Balkan; Graf et al. 2008), and that the endemic status of the three species clearly defined their non-overlapping sampling ranges. Information on the morphology of the larvae is given, and the most important diagnostic features are illustrated.

## Introduction

In Europe, the genus *Apataniana* was unknown until 1987 when Malicky discovered a large number of unknown Apataniidae larvae in a small stream at 1200 m a.s.l. in the Ossa mountains at the southern side of the Olymp massif; at the same location, a number of adults were caught in light traps and described as *Apataniana hellenica*. The second new species was *Apataniana vardusia* from a small stream above the tree line at 1750 m above sea level in the Vardusia mountains in Central Greece (Malicky 1992). Finally, *Apataniana stropones* was discovered in August 1993 at the eastern slopes of the Dirfis mountains on the island of Euböa (Malicky 1993). Interestingly, the closest relatives of the three Greek *Apataniana* species are reported from Asia Minor (Turkey; *Apataniana borcka* Sipahiler, 1996) and Asia (Kyrghyzstan: *Apataniana rauschorum* Malicky, 1999; Tadzhikistan: *Apataniana pamirensis* Mey and Levanidova, 1989; Kazakhstan, Tadzhikistan, Uzbekistan: *Apataniana cornuta* Ivanov, 1991, *Apataniana elongata* (McLachlan, 1875), *Apataniana propria* Mey, 1986; Mongolia, Tibet: *Apataniana impexa* Schmid, 1968, *Apataniana hutchinsoni* Mosely, 1936, *Apataniana spinosa* Yang & Tao, 2011; Indian Himalaya: *Apataniana charadija* Schmid, 1968; Russia: vicinity of lake Baikal: *Apataniana bulbosa* Martynov, 1918; Chukotski Peninsula: *Apataniana tschuktschorum* Levanidova, 1979) (Mey and Levanidova 1989; Malicky 2005b; Morse 2015).

Mey and Levanidova (1989) proposed three species groups, based on phylogenetic and biogeographical considerations (*impexa*, *bulbosa*, and *elongata* group) and presented morphological information for characterizing genus *Apataniana* in the larval stage which is morphologically very close to *Apatania* Kolenati, 1848. The same authors also provided a first key for larval *Apataniana* including *Apataniana hellenica*, *Apataniana pamirensis*, *Apataniana tschuktschorum*, *Apataniana elongata* and *Apataniana impexa*. With our descriptions, proposed here, the identification of all three European *Apataniana* larvae is now possible even without adults.

## Material and methods

The larval material was sampled by Hans Malicky at the following locations and dates: *Apataniana hellenica* Malicky, 1987: Ossa mountains, ‘*Apataniana* stream’ (22°42'E, 39°50'N, 1200 m a.s.l.) on 28 July1991; *Apataniana stropones* Malicky, 1993: Euböa, Dirfis mountains near Stropones (23°53'E, 38°36'N, 700–900 m a.s.l.) on 24 May1974 and 5 August 1993; *Apataniana vardusia* Malicky, 1992: Vardusia mountains above tree line, Central Greece (22°08'E, 38°42'N, 1750 m a.s.l.) on 22 October 1991.

A hand net was used to collect larvae, and light trapping obtained the adult material of the three *Apataniana* species which also included the holo- and paratypes for the species descriptions (details on the latter are given by Malicky 1987, 1992, 1993, 2005b). The material was preserved in 70% ethanol. A Nikon SMZ 1500 binocular microscope with DS-Fi1 camera and NIS-elements D 3.1 image stacking software for combining 7–38 frames in one focused image were used to study and photograph the larvae.

Species association was enabled by the fact that the three European *Apataniana* larvae are the only Apataniidae species recorded in European ecoregion 6 (Hellenic Western Balkan; Graf et al. 2008) and that the endemic status of the three species restricts their distribution range, preventing confusion with other closely related species. The three 5th instar larvae of each species used for the descriptions are deposited in the collection of J. Waringer (Vienna, Austria); further larval, pupal and adult material is stored in the collection of H. Malicky (Lunz am See, Austria). We used the morphological terminology by Wiggins (1998), Wallace et al. (2003) and Waringer and Graf (2011).

## Results

### Apataniana stropones

Taxon classificationAnimaliaTrichopteraApataniidae

Malicky, 1993

#### Description of the 5th instar larva.

**Diagnosis.** Setae at anterior edge of pronotum long, tapering and with flexuous tips; setal transversal band at 1st abdominal dorsum continuous; 2nd tarsal claw shorter than half tarsal length; central submentum sides converging.

**Biometry.** Body length of 5th instar larvae ranging from 4.9 to 5.0 mm, head width from 0.73 to 0.74 mm (n= 3).

**Figures 1–4.**
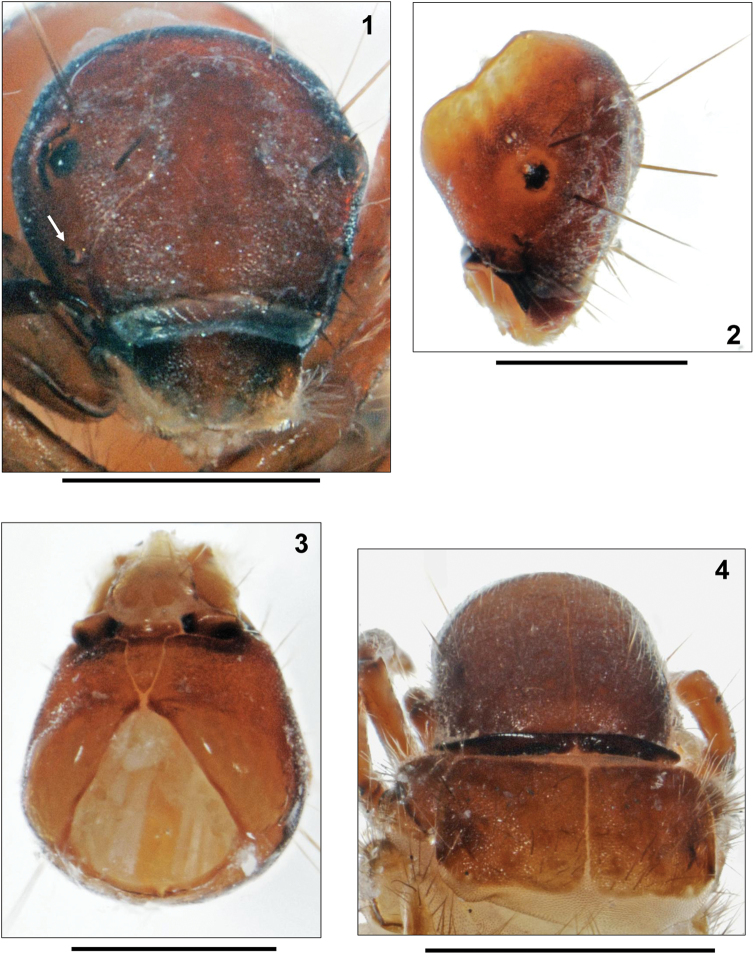
*Apataniana stropones* Malicky 1993, 5th instar larva. **1** Head, frontal view (arrow: antenna) **2** Head, right fronto-lateral view **3** Head, ventral view **4** Pro- and mesonotum, dorsal view. Scale bars: 0.5 mm (except Fig. **4**: 1 mm).

**Head.** Head capsule with dense cover of microspinules, roundish in shape and hypognathous (Figs 1–3). Coloration medium to dark brown; paler areas around eyes and around foramen occipitale (Figs 1, 2). Muscle attachment spots on frontoclypeus and parietalia small and indistinct (Figs 1, 2). In addition to complete set of primary setae, head capsule with many short, pale and almost translucent secondary setae (Fig. 2). Frontoclypeus bell-shaped, with narrow central constriction (Fig. 1). Antennae situated halfway between eye and anterior head margin (Fig. 1, arrow), short, each consisting of 1 short cylindrical base and 1 prominent lateral seta. Labrum dark brown, with setal brush at anterolateral corners originating from whitish pads of soft cuticle. Submentum wedge-shaped, sclerite almost as wide as long, convex-sided at center; light brown with medium brown pre-apical transverse band. Postgenal suture less than 20% of apotome length. Scraper-type mandibles (as in Fig. 15) black, brownish terminally, elongated quadrangular and without terminal teeth along cutting edge (as in Fig. 15).

**Figures 5–11.**
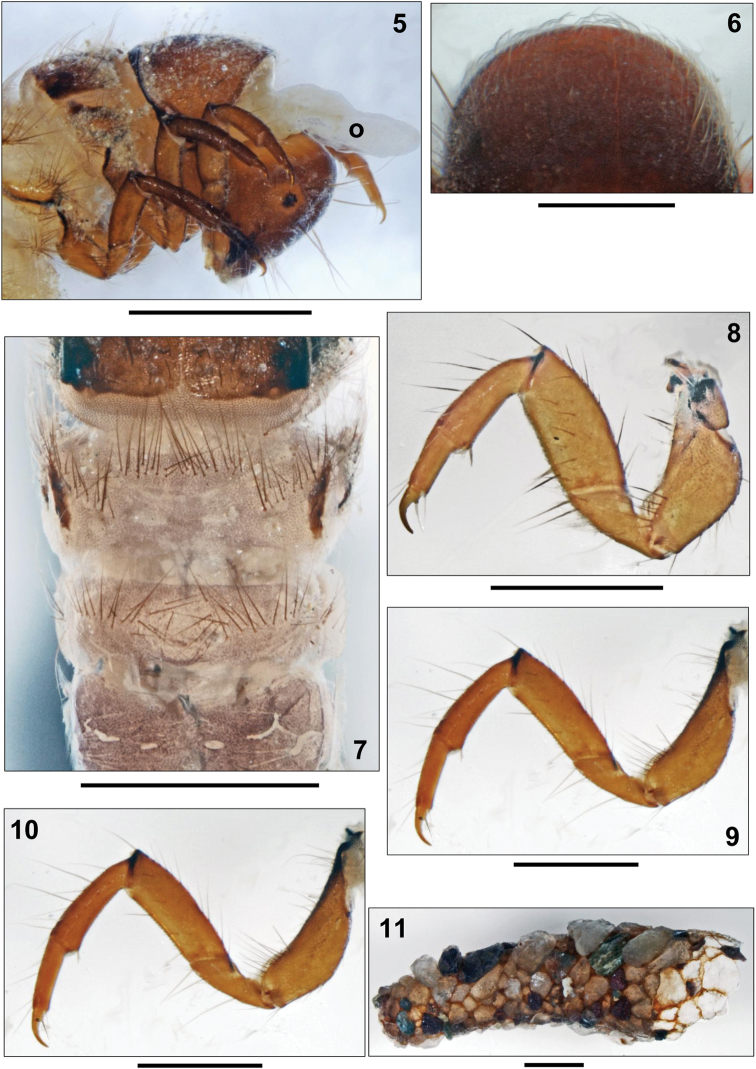
*Apataniana stropones* Malicky 1993, 5th instar larva. **5** Head and thorax, right lateral view (o: osmeterium) **6** Pronotum, dorsal view **7** Metathorax and anterior abdominal segments, dorsal view **8** Left foreleg, posterior face **9** Left midleg, posterior face **10** Left hind leg, posterior face **11** Larval case, right lateral view. Scale bars: 0.5 mm (except Figs **5**, **7**, **11**: 1 mm).

**Figures 12–18.**
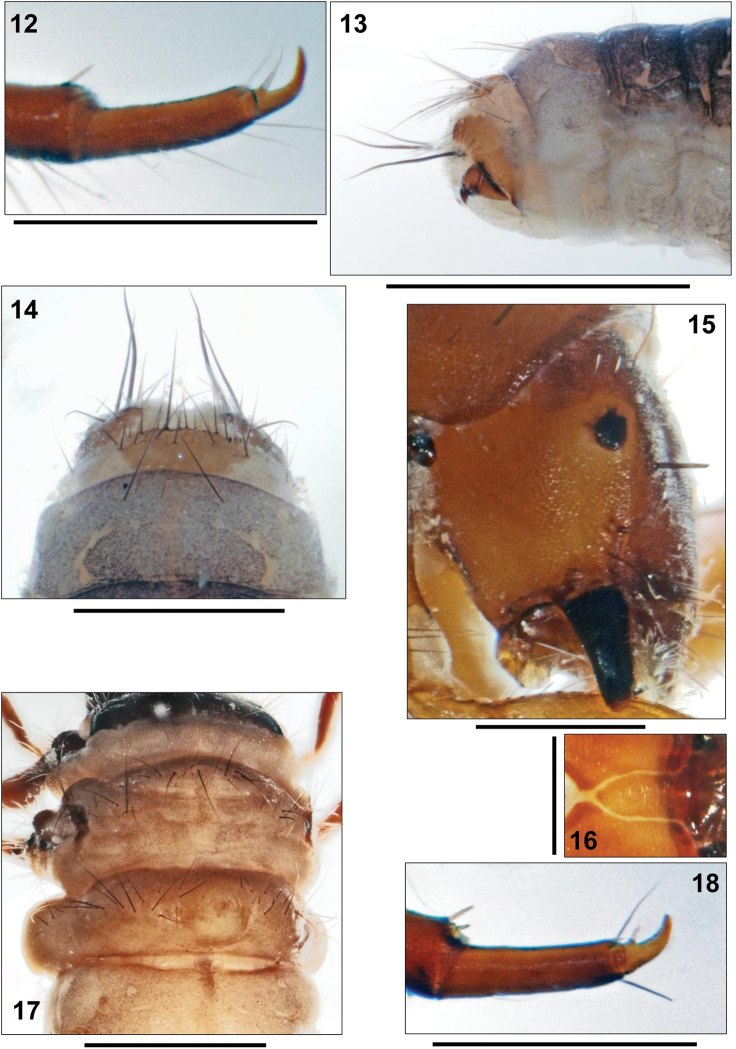
**12–14** *Apataniana stropones* Malicky 1993, 5th instar larva. **12** Mid tarsus and claw, anterior face **13** Tip of abdomen, right lateral view **14** Ninth abdominal dorsum, dorsal view **15–18** *Apataniana hellenica* Malicky 1987, 5th instar larva. **15** Head, right lateral view **16** Submentum **17** Metathorax and anterior abdominal segments, dorsal view **18** Mid tarsus and claw, anterior face. Scale bars: 0.5 mm (except Figs **12**, **16**, **18**: 0.25 mm and Figs **13**, **17**: 1 mm).

**Thorax.** Pronotum medium brown, surface densely granulated, posterior margins thickened and darkly striped (Figs 4, 5). Pronotal transverse groove lacking, as typical for Apataniidae larvae (Figs 5, 6). In profile, pronotum slightly rounded (Fig. 5). Pronotal surface densely covered by pale, translucent, tapering setae with flexuous tips, especially along the anterior border (Fig. 6); in addition 30–40 longer setae present on each pronotal half (Figs 4, 5). As in other Apataniidae larvae, a defensive gland is situated beneath the pronotal sclerites. In all three European *Apataniana* larvae, an additional Y-shaped appendix is present which can be extended at the cervix between pronotum and head (=osmeterium; Fig. 5o); when threatened, the gland is able to produce a mix of up to 40 fatty acids as a defense agent against predacious invertebrates (Wagner et al. 1990). Pentangular prosternite densely covered by microspinules, very pale and indistinct in its anterior and lateral sections; along posterior border with light brown transverse band; with distinct, light brown and triangular lateral sclerites. Prosternal horn present. Mesonotum completely covered by 2 light brown sclerites; their anterior, lateral and postero-lateral margins darker sclerotized; mesonotal surface with medium brown muscle attachment spots (Fig. 4). Metanotum partially covered by only 1 pair of yellowish lateral sclerites with anterior groups of approximately 20 setae per sclerite and dark brown muscle attachment spots; anterior and posterior metanotal sclerites completely lacking and replaced by groups of setae: 15–20 at each anterior and 15–18 at each posterior metanotal position (Fig. 7). Legs yellowish to light brown with numerous setae on coxae, trochanters and femora; tibiae and tarsi with only a small number of setae (Figs 8–10). Femora each with more than 1 proximodorsal seta. Coxa, femur and tibia of each foreleg wider than those of mid- and hind legs. Additional setae present at anterior and posterior faces of all femora. Setae lacking at distal sections of trochanter on all legs. Mid tarsal claw shorter than half tarsal length; in addition, tarsal claw seta long and almost reaching tip of tarsal claw (Fig. 12).

**Abdomen.** 1st abdominal segment with 1 dorsal and 2 lateral fleshy protuberances densely covered by microspinules. Setal transversal band at 1st abdominal dorsum continuous at center, consisting of 45–60 setae (Fig. 7); dorsal of each lateral protuberance, an additional group of 7–10 setae is present. Ventral section of lateral protuberances and 1st abdominal sternum with continuous field of setae with basal sclerites minute and inconspicuous; total setal number is 130–150.

8th abdominal dorsum with 14–18 posterodorsal setae; several posterolateral setae on each half of 9th abdominal dorsum. All gills single filaments. Dorsal gills present at most from 2nd segment (postsegmental position) to 4th segment (postsegmental position). Ventral gills ranging from 2nd (postsegmental) to 6th segment (postsegmental). Lateral gills lacking. Lateral fringe extending from start of 2nd to mid 8th abdominal segment. Dorsal of lateral fringe a small number of forked lamellae is present per segment (as in Figs 20, 21).

**Figures 19–26.**
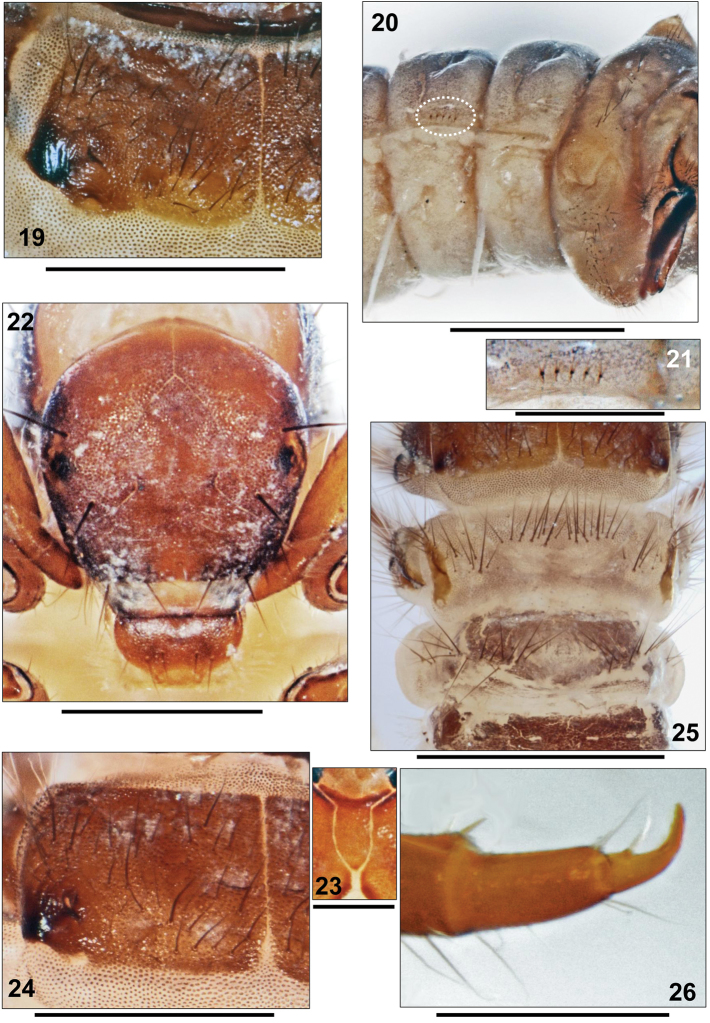
**19–21** *Apataniana hellenica* Malicky 1987, 5th instar larva. **19** Left mesonotum, dorsal view **20** Anterior abdominal segments, right lateral view (dotted oval: forked lamellae) **21** Detail of forked lamellae situated dorsal of lateral fringe **22–26.** *Apataniana vardusia* Malicky 1992, 5th instar larva. **22** Head, frontal view **23** Submentum **24** Left mesonotum, dorsal view **25** Metathorax and anterior abdominal segments, dorsal view **26** Mid tarsus and claw, anterior face. Scale bars: 0.5 mm (except Figs **20**, **25**: 1 mm and Figs **23**, **26**: 0.25 mm).

Light brown sclerite on 9th abdominal segment semicircular, with light muscle attachment spots; with 26–33 setae, 6 long and the remainder short to medium in length (Fig. 14). Anal prolegs of the limnephilid type, light brown and with dark brown bar at anterodorsal border of claw base. Anal proleg lateral sclerite with 5 setae along posterior edge (Fig. 13). Anal claws dark brown, with tiny dorsal accessory hook.

**Case.** Fifth instar larval case 5.4–5.5 mm long (n= 3), curved, tapering posteriorly (width at anterior opening 1.8–2.0 mm and at posterior opening 0.9–1.1 mm), consisting of mineral particles of varying size, sometimes mixed with larger particles attached mostly laterally, thereby creating wing-like structures (Fig. 11).

### Apataniana hellenica

Taxon classificationAnimaliaTrichopteraApataniidae

Malicky, 1987

#### Description of the 5th instar larva

(all morphological characters identical to those of *Apataniana stropones* except as noted below). **Diagnosis.** Setal transversal band at 1st abdominal dorsum interrupted at center; central submentum parallel-sided.

**Biometry.** Body length of 5th instar larvae ranging from 6.4 to 6.9 mm, head width from 0.76 to 0.83 mm (n= 3).

**Head.** Head reddish brown with paler, wedge-shaped areas from eyes to mandible bases and around anterior border of head capsule (Fig. 15). Labrum medium to dark brown. Submentum narrower than in *Apataniana stropones*, wedge-shaped, parallel-sided at center (Fig. 16); light to yellowish brown, with dark brown anterior, broadened section and medium brown posterior tip.

**Thorax.** Pronotum dark brown. Pentangular prosternite with medium brown transverse band; lateral sclerites indistinct. Mesonotum medium brown, posterolateral corners black (Fig. 19). Lateral sclerites with anterior groups of 10–20 setae per sclerite; anterior and posterior metanotal sclerites completely lacking and replaced by groups of setae: 10–28 at each anterior and 10–15 at each posterior metanotal position (Fig. 17). Mid tarsal claw shorter than half tarsal length (Fig. 18).

**Abdomen.** Setal transversal band at 1st abdominal dorsum interrupted at center, consisting of 30–40 setae (Fig. 17); setal group dorsal of lateral protuberance with 4–7 setae each. Ventral section of lateral protuberances and 1st abdominal sternum with continuous field of setae with basal sclerites minute and inconspicuous; total setal number is 120–140.

Dorsal gills present at 3rd segment (postsegmental position), ventral gills ranging from 2nd (postsegmental) to 6th segment (postsegmental). Lateral gills lacking.

**Case.** Fifth instar larval case 8.3^_^8.7 mm long (n= 3), curved, tapering posteriorly (width at anterior opening 2.5–4.9 mm and at posterior opening 1.5–1.8 mm).

### Apataniana vardusia

Taxon classificationAnimaliaTrichopteraApataniidae

Malicky, 1992

#### Description of the 5th instar larva

(all morphological characters identical to those of *Apataniana stropones* except as noted below). **Diagnosis.** Setal transversal band at 1st abdominal dorsum interrupted at center; 2nd tarsal claw longer than half tarsal length; central submentum parallel-sided.

**Biometry.** Body length of 5th instar larvae ranging from 4.4 to 5.3 mm, head width from 0.70 to 0.73 mm (n= 3).

**Head.** Head medium brown (Fig. 22). Submentum narrower than in *Apataniana stropones*, wedge-shaped, parallel-sided at center (Fig. 23); light to yellowish brown, with medium brown anterior, broadened section and pale posterior tip. Postgenal suture approximately 30% of apotome length (Fig. 23).

**Thorax.** Pronotal surface medium brown, with pale, small, roundish muscle attachment spots. In addition to dense cover of pale, translucent, tapering setae with flexuous tips 20–30 longer setae present on each pronotal half. Pentangular prosternite pale, with medium brown posterior transverse band; lateral sclerites indistinct. Mesonotum medium brown, posterolateral corners black; near anteromedian corner of this black spot with ear-like porus (Fig. 24). Lateral sclerites with anterior groups of approximately 10 setae per sclerite; anterior and posterior metanotal sclerites completely lacking and replaced by groups of setae: 5–10 at each anterior and 5–10 at each posterior metanotal position (Fig. 25). Mid tarsal claw longer than half tarsal length (Fig. 26).

**Abdomen.** Setal transversal band at 1st abdominal dorsum interrupted at center, consisting of 30–40 setae (Fig. 25); setal group dorsal of lateral protuberance with 4–7 setae each. Ventral section of lateral protuberances and 1st abdominal sternum with continuous field of setae with basal sclerites minute and inconspicuous; total setal number is 110–120. 8th abdominal dorsum with 18–20 posterodorsal setae. Light brown sclerite on 9th abdominal segment with 22–34 setae, 6 long and the remainder short to medium in length.

**Case.** Fifth instar larval case 6.3–6.4 mm long (n= 3), curved, tapering posteriorly (width at anterior opening 2.4–2.5 mm and at posterior opening 1.3–1.4 mm).

### Synoptic key for the European *Apataniana* larvae (final instars)

Larval Apataniidae share the following set of morphological characters (Pitsch 1993, Solem 1985, Wallace et al. 2003, Waringer and Graf 2011, Wiggins 1998): transportable case present (Fig. 11); sclerites present on pro-, meso- and metanota (Fig. 5); no transverse rim at the anterior 3rd of the pronotum (Fig. 5); pronotum and mesonotum completely covered by 2 sclerites in close contact, separated by an unbranched longitudinal suture (Fig. 4); median and posterior metanotal sclerites reduced and represented only by setal groups (Fig. 7); prosternal horn present; antennae situated halfway between eye and anterior head margin (Fig. 1, arrow); scraper-type mandibles without terminal teeth along cutting edge (Fig. 15); submentum wedge-shaped (Fig. 3); head with many secondary setae (Fig. 2); fleshy protuberances present laterally and dorsally on the 1st abdominal segment (e.g., Fig. 7); all gills consisting of single filaments (Fig. 20).

In the framework of the larval key to European Apataniidae of Waringer et al. (2015) the three European species of genus *Apataniana* can be easily integrated by using the morphology of setae at the anterior edge of pronotum which are long, tapering and with flexuous tips (Fig. 6) and by the fact that the genus is restricted to the Hellenic Western Balkan (Greece).

Within the trio of *Apataniana* species, the setal transversal band at 1st abdominal dorsum is interrupted at center in *Apataniana vardusia* and *Apataniana hellenica* (Figs 17, 25) but continuous in *Apataniana stropones* (Fig. 7). The former species pair can be separated by the mid tarsal claw/tarsus ratio (Figs 18, 26), and by differences in head capsule width (Table 1). Submentum morphology (Figs 3, 16, 23) provides additional characters for identification of the three species.

**Table 1.** Synopsis of characters separating the currently known European *Apataniana* larvae (5th instars; Trichoptera: Apataniidae) (Malicky 1987, 1992, 1993, 2004, 2005a, b; Graf et al. 2008).

Species/ character	Setal transversal band at 1st abdominal dorsum interrupted at center (Fig. 17)?	2nd tarsal claw shorter than half tarsal length (Fig. 12)?	Submentum almost parallel-sided at mid section (Fig. 16)?	Head width (mm)	Distribution
*Apataniana vardusia*	yes	no	yes	0.70–0.73	endemic of Vardusia mountains, Central Greece
*Apataniana hellenica*	yes	yes	yes	0.76–0.83	endemic of Ossa mountains, Eastern Thessalia, Greece
*Apataniana stropones*	no	yes	no	0.73–0.74	endemic of Dirfis moutains, Euböa, Greece

### Ecology, phenology and distribution

With respect to distribution, *Apatania hellenica* is an endemic species of the Ossa mountains in Eastern Thessalia, *Apataniana stropones* is endemic to the Dirfis moutains on the island of Euböa and *Apataniana vardusia* is an endemic species of the Vardusia mountains in Central Greece. The larvae of the three *Apataniana* species inhabit small brooklets with low water temperatures (Malicky 1987, 1992, 1993, 2005b, 2014a, b). *Apataniana vardusia* was sampled above the tree line at an altitude of 1750 m a.s.l. where water temperature in the source of the brook was, as common for this altitude, 5.7°C in October and 5.7°C at the end of May. In the other two species, typical habitats are situated at lower altitudes: *Apataniana hellenica* lives in the spring area and a short distance downstream of a brook in the Ossa Mountains situated at 1200 m a.s.l. (photograph in Malicky 2014b, p. 190) where water temperature was 5.4°C in May, 5.5°C at the end of July, and 5.5°C on the onset of October. Only 300 m downstream of this spot, the water temperature was 6.1–6.8°C at the end of May, 6.6–6.8°C at the beginning of June, 7.8–10.0°C at the end of July, and 7.6–8.0°C at the onset of October. A few specimens were occasionally found in other nearby springbrooks. *Apataniana stropones* was collected in spring brooklets at the northern slope of Mt. Dirfis on the island of Euböa, at about 700 to 900 m a.s.l., where the water temperatures were 10.9°C in May, 10.5°C in June, and 10.2°C in August. A similar brook without *Apataniana* only 5 km eastward had water temperatures of 13.6–15.5°C in June, which is the usual temperature to be expected in Greek mountains at these elevations. Obviously, the three species are cold stenothermous. We have no explanation for the low water temperatures of the spring areas inhabited by *Apataniana hellenica* and *Apataniana stropones*. Karst phenomena are unknown in these areas, as the underground consists of various kinds of silicate rocks, and the area is covered by dense natural forest. Both areas are situated at the slopes of the mountains close to the sea. Dense cloud banks may be sometimes seen at mid-slope, often for many hours during fine weather, which may contribute to the low water temperatures of these spring locations.

As in the other known larvae of Apataniidae, the mandibles in the three Greek *Apataniana* species take the shape of scraper blades and lack terminal teeth along their cutting edges; larvae graze autotrophic biofilm and epilithic algae.

Adults of *Apataniana hellenica* were found in June and July, but not in May and October; ultimate and penultimate larval instars were collected in May, June, July and October, mature pupae at the end of July. Therefore, the adults are on the wing between June and September, with one generation per year, but the presence of many ultimate instar larvae in October may be an indication that some individuals may need more than one year for their development. The phenology of *Apataniana stropones* is similar. Only one male was collected at the end of June, but at the onset of August not only high numbers of adults but also mature and immature pupae and many ultimate and penultimate instar larvae were observed. In October no adults were present. In *Apataniana vardusia*, on the other hand, the adult stage is entered later in the year. At the end of May, many larvae but no adults were found. At the end of October, adults, many fresh egg clutches, many empty cases and some ultimate and penultimate larvae were observed.

The three Greek *Apataniana* species are confined to extremely small areas. *Apataniana vardusia* was only found in one spring brook some metres long. *Apataniana hellenica* and *Apataniana stropones* are restricted to a couple of brooklets in distances of several hundred metres. These tiny distribution ranges and the extremely low water temperatures of their habitats earmark the trio of Greek *Apataniana* species as glacial relics. It would be no surprise to detect some more closely related species in other mountain massifs in Greece.

**Figure 27.**
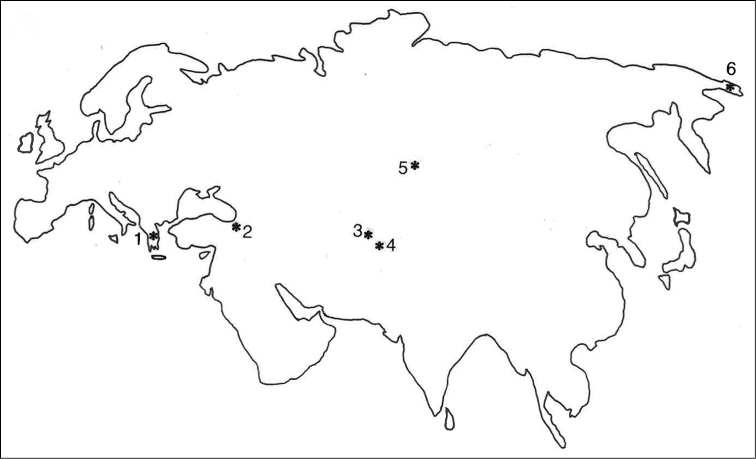
Distribution of *Apataniana* species of the *bulbosa* group: **1** *Apataniana hellenica*, *Apataniana stropones*, *Apataniana vardusia* **2** *Apataniana borcka* **3** *Apataniana rauschorum* **4** *Apataniana pamirensis* **5** *Apataniana bulbosa* **6** *Apataniana tschuktschorum*.

The three species described above belong to the *Apatania bulbosa* group (Mey and Levanidova 1989). The other known species of this group include *Apataniana borcka* from the Caukasus (Turkey, Province of Artvin), *Apataniana rauschorum* from Kyrghyzstan, *Apataniana bulbosa* from the Sajan Mountains, *Apataniana pamirensis* from Tadzhikistan, and *Apataniana tschuktschorum* from the Chukotski Peninsula in the extreme east of Siberia (Fig. 27). All of these species are extremely relictary, and to the best of our knowledge they live in cold streams. They are obviously Pleistocene relics, but the long distances between their areas, averaging thousands of kilometres, suggest that they are relics from one of the earlier (about 20) glaciation periods. Relics from the last (Würm) glaciation period are more often found, but they usually have other patterns of distribution (Malicky 1988). A parallel case in the European fauna is *Apatania volscorum* Moretti, 1988, restricted to a karst area south of Rome at 300 m a.s.l. It is only known from cold springbrooks with water temperatures of 9.6–10.2°C throughout the year. Its next relative, *Apatania ulmeri* Schmid, 1950, is restricted to the Sajan Mountains (near Lake Baikal) in a distance of more than 5000 kilometres away (Bicchierai and Moretti 1988; Corallini and Moretti 1988; Malicky 2005b; Moretti et al. 1988; Spinelli and Moretti 1988).

## Supplementary Material

XML Treatment for Apataniana stropones

XML Treatment for Apataniana hellenica

XML Treatment for Apataniana vardusia
